# Oncological impact of unexpected horizontal tumour spread in oesophagogastric junction cancer

**DOI:** 10.1093/bjsopen/zraf119

**Published:** 2025-10-07

**Authors:** Qingjiang Hu, Manabu Ohashi, Motonari Ri, Rie Makuuchi, Tomoyuki Irino, Masaru Hayami, Takeshi Sano, Souya Nunobe

**Affiliations:** Department of Gastroenterological Surgery, Cancer Institute Hospital, Japanese Foundation for Cancer Research, Tokyo, Japan; Department of Gastroenterological Surgery, Cancer Institute Hospital, Japanese Foundation for Cancer Research, Tokyo, Japan; Department of Gastroenterological Surgery, Cancer Institute Hospital, Japanese Foundation for Cancer Research, Tokyo, Japan; Department of Gastroenterological Surgery, Cancer Institute Hospital, Japanese Foundation for Cancer Research, Tokyo, Japan; Department of Gastroenterological Surgery, Cancer Institute Hospital, Japanese Foundation for Cancer Research, Tokyo, Japan; Department of Gastroenterological Surgery, Cancer Institute Hospital, Japanese Foundation for Cancer Research, Tokyo, Japan; Department of Gastroenterological Surgery, Cancer Institute Hospital, Japanese Foundation for Cancer Research, Tokyo, Japan; Department of Gastroenterological Surgery, Cancer Institute Hospital, Japanese Foundation for Cancer Research, Tokyo, Japan

**Keywords:** distal margin, proximal margin, prognostic factor, oesophageal, gastric cancer, survival

## Abstract

**Background:**

Unexpected horizontal tumour spread towards the proximal and distal margins complicates the assessment of surgical margins in oesophagogastric junction (OGJ) cancer. Its impact on oncological outcomes remains unclear.

**Methods:**

This study retrospectively analysed patients with OGJ adenocarcinoma undergoing proximal or total gastrectomy. Unexpected horizontal tumour spread was measured as the discrepancy between gross and pathological margins proximally (ΔPM) and distally (ΔDM). Clinicopathological features, recurrence-free survival (RFS), and overall survival (OS) were evaluated based on ΔPM and ΔDM.

**Results:**

Based on cut-off values identified by time-dependent receiver operating characteristic curve analysis (ΔPM, 8 mm; ΔDM, 3 mm) in 197 patients, patients were classified into four groups: short; long ΔPM; long ΔDM; and both long ΔPM and ΔDM (both-long). RFS was best in the short group and worst in the both-long group. The long ΔPM and long ΔDM groups had intermediate and comparable RFS. Subsequently, patients were categorized into two groups: a short group and a long group, which included patients in the long ΔPM, long ΔDM, and both-long groups. The type of infiltrative growth and postoperative recurrence were significantly associated with the long group. Moreover, the long group had significantly worse RFS and OS than the short group. Multivariate Cox regression analyses identified the long group as an independent risk factor for both RFS and OS. Patients in the long group with clinical lymph node metastasis or tumours located in the proximal 2-cm segment of the OGJ, predominantly in the proximal rather than distal 2-cm segment of the OGJ, or equal involvement in both areas had markedly worse survival outcomes.

**Conclusion:**

Unexpected horizontal tumour spread, represented by ΔPM and ΔDM, is a strong predictor of poor survival and recurrence in OGJ cancer. Intraoperative assessment of ΔPM and ΔDM using frozen section analysis may be useful in guiding additional resections, particularly when combined with other predictive factors.

## Teaser

Unexpected horizontal tumour spread, proximally and distally, significantly predicts poor survival and recurrence in oesophagogastric junction cancer. Intraoperative assessment of the discrepancy between gross and pathological margins proximally (ΔPM) and distally (ΔDM), combined with clinical factors, may help surgeons optimize surgical strategies.

## Introduction

The incidence of oesophagogastric junction (OGJ) cancer has been increasing worldwide^1–3^. OGJ cancer, characterized by its unique anatomical location and biological behaviour, poses significant challenges in surgical and oncological management^4^. Proximal gastrectomy (PG) is a recommended treatment option^5^; however, to achieve complete resection while preserving as much of the remnant stomach as possible, careful attention must be given to both the proximal and distal margins. In addition, OGJ cancer is considered to have worse survival than gastric cancer^6^.

One of the critical issues in PG for OGJ cancer is the occurrence of unexpected horizontal tumour spread (microscopic submucosal spread), where the tumour infiltrates beyond the grossly visible boundaries into the submucosal layers both proximally and distally. This phenomenon often leads to discrepancies between the gross and pathological margins on both the oral and aboral sides. Failure to account for such discrepancies may result in positive surgical margins, which should be considered for additional resection to achieve pathologically negative margins because pathologically positive margins are strongly associated with poor survival outcomes^7^.

The discrepancy between pathological and gross resection margins has been investigated for several years and the minimum resection lengths required for various types of gastrectomy have been determined. To express and clarify discrepancies between pathological and gross resection margins, their lengths were defined as ΔPM and ΔDM on the proximal and distal sides, respectively, representing how long the pathological tumour boundary unexpectedly spreads beyond the gross boundary towards the proximal or distal resection stump^8–11^. ΔPM and ΔDM have been assessed in patients with gastric cancer or OGJ cancer who underwent total gastrectomy (TG), distal gastrectomy (DG), or PG, and the recommended lengths for PM and DM have been reported previously. Furthermore, it was recently reported that ΔPM was strongly associated with survival outcomes in patients with gastric cancer or OGJ cancer who underwent curative total gastrectomy^12^. Therefore, it was hypothesized that both ΔPM and ΔDM may affect tumour progression and influence survival outcomes in patients with OGJ cancer.

This study investigated the oncological impact of unexpected horizontal tumour spread (ΔPM and ΔDM) in patients with OGJ cancer who underwent curative resection. Clinicopathological features and survival outcomes were analysed in relation to ΔPM and ΔDM values. The findings of this study may help identify patients at high risk of recurrence and poor prognosis while improving intraoperative decision-making.

## Methods

### Patient selection

**Fig. 1:**
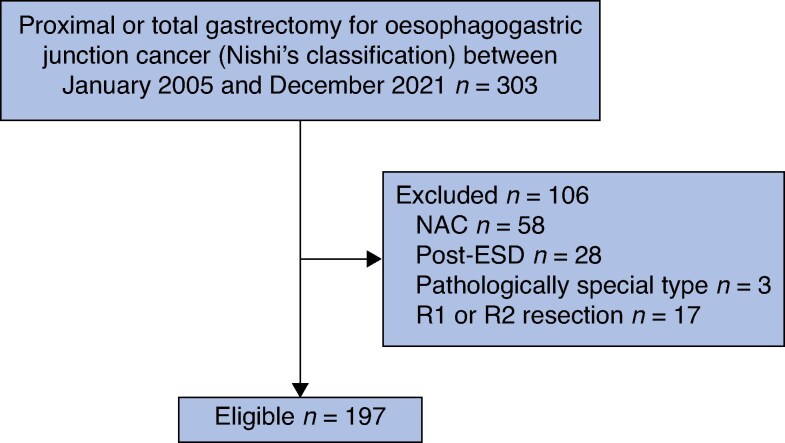
Study flow chart NAC, neoadjuvant chemotherapy; ESD, endoscopic submucosal dissection.

Patients with OGJ adenocarcinoma who underwent PG or TG via a transabdominal approach between January 2005 and December 2021 at the Department of Gastroenterological Surgery, Cancer Institute Hospital of the Japanese Foundation for Cancer Research, were retrospectively included in this study. OGJ cancer was defined according to Nishi’s classification^4^, which describes a tumour with its epicentre located within 2 cm above (E) or below (G) the OGJ, with the dominant area of invasion described first (for example EG, GE, or E=G if both areas equally involved). Patients who underwent endoscopic submucosal dissection or who received neoadjuvant chemotherapy were excluded. In addition, patients with a pathologically positive proximal margin (PM) or distal margin (DM) who did not undergo additional resection were excluded because the length of the PM or DM could not be measured. Only patients who underwent R0 resection were enrolled in this study (*Fig. 1*).

Growth type was classified according to the Japanese Classification of Gastric Carcinoma 3rd English edition (JCGC)^4^. Patients with macroscopic type 0 tumours were classified as having a superficial growth type, which included all patients classified as clinical tumour depth (cT) 1 and some patients classified as cT2–4. Patients with macroscopic type 1 and 2 tumours were classified as having an expansive growth type, and those with type 3 and 4 tumours were classified as having an infiltrative growth type. For patients with type 5 tumours, preoperative findings were re-evaluated, and tumours were classified as expansive or infiltrative growth.

Patients were divided into two histopathological groups: differentiated and undifferentiated. The differentiated type included well or moderately differentiated adenocarcinoma and papillary adenocarcinoma, whereas the undifferentiated type comprised solid and non-solid types of poorly differentiated adenocarcinoma, signet ring cell carcinoma, and mucinous adenocarcinoma. Clinical and pathological stages were determined based on the JCGC, as outlined previously^13^.

Data were retrieved from a prospectively developed database.

This study was approved by the Ethics Committee of the Cancer Institute Hospital of Japan Foundation for Cancer Research (Approval no. 2023-GB-092). Patient consent was obtained through an opt-out process.

### Surgical procedures

All patients enrolled in this study underwent PG or TG with D1+ lymphadenectomy for cT1 disease or D2 lymphadenectomy for cT1N(+) or cT2–4 disease, in accordance with the 2021 Japanese gastric cancer treatment guidelines^5^. In Japan, the standard surgical procedure for OGJ cancer has traditionally been total gastrectomy. However, in recent years, evidence has emerged indicating that there is little to no lymph node metastasis around the distal part of the stomach in OGJ cancer^14^. As a result, proximal gastrectomy, which preserves the distal stomach, has become the first-line surgical option for OGJ cancer.

### Measurement of gross PM and DM lengths

As previously reported^9,13^, fresh specimens were opened longitudinally, and the lymph nodes were removed for pathological examination; the specimen was then naturally spread out and pinned to a flat board with the mucosal side facing upward.

The clinical tumour boundary was determined on the basis of at least two visual and tactile examinations by the surgeons, referring to the findings of preoperative endoscopy and a barium meal study. Preoperative endoscopy is routinely conducted with standard observation, complemented by indigo carmine spraying and narrow-band imaging to enhance lesion visualization. When the boundary could not be definitively determined, endoscopists or pathologists were consulted.

Gross findings were written on an individual’s chart and the specimen was finally photographed against a scale. The clinical oesophageal invasion (cOI) length, gross PM length, and gross DM length were referred to on the chart and additionally measured using the photographs to confirm them.

### Postoperative treatment and follow-up

After surgery, the indication for adjuvant chemotherapy was based on the results of the ACTS-GC^15^, CLASSIC^16^, and START-II studies^17^. Briefly, patients with pathological stage II or III disease after surgery underwent S-1 (tegafur, 5-chloro-2, 4-dehydroxypyridine, and potassium oteracil) monotherapy for 1 year, capecitabine/S-1 plus oxaliplatin for 6 months, or docetaxel plus S-1 for 1 year. Blood tests, including carcinoembryonic antigen and carbohydrate antigen 19-9 levels, were performed at least once every 3 months, and enhanced thoraco-abdominal computed tomography was performed every 3–6 months.

### Pathological examination and determination of pathological PM and DM lengths

Each specimen was fixed in 10% buffered formalin solution for 48 hours. Specimens were cut into serial sections (at 5-mm intervals) and subsequently evaluated pathologically according to the JCGC^4^. After pathological evaluation, the tumour was mapped using the images of the fixed specimen. Using this mapping in the pathological reports, the locations of the pathological proximal boundary, the pathological distal boundary, the pathological PM length, and the pathological DM length were determined. The pathological report was prepared by board-certified pathologists as part of routine clinical practice. The pathological proximal and distal boundaries included not only continuous mucosal or submucosal lesions but also intermittent lesions, such as lymphatic or vascular invasion.

### Definition of parameters

**Fig. 2:**
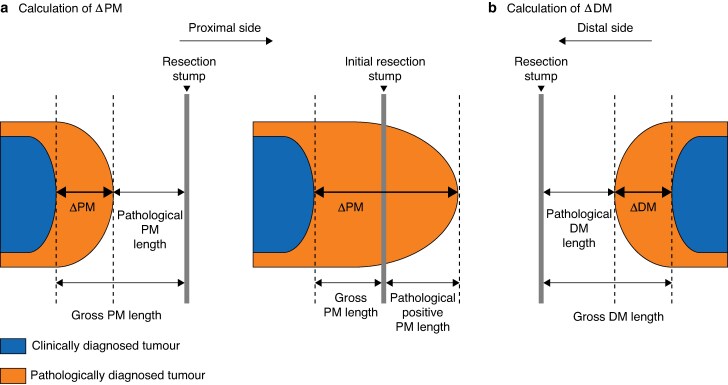
Calculation of ΔPM and ΔDM **a** In a pathologically negative PM (left), ΔPM is calculated as follows: ΔPM = gross PM length—pathologically negative PM length. When a PM is pathologically positive in the initial transection but negative in the additional transection (middle), tumour lesions are present not just in the initially resected specimen. Thus, the ΔPM is calculated as follows: ΔPM = gross PM length in the initial specimen + pathologically positive PM length in the additional specimen. **b** In a pathologically negative DM, ΔDM is calculated as follows: ΔDM = gross DM length—pathologically negative DM length. ΔPM, discrepancy between gross and pathological margins proximally; PM, proximal margin; ΔDM, discrepancy between gross and pathological margins distally; DM, distal margin.

Consistent with previous studies^8–13,18^, ΔPM was defined as the discrepancy between the gross and pathological proximal boundary of the tumour, which indicates how far the pathological tumour boundary extends beyond the gross boundary towards the proximal resection stump. ΔDM was defined the discrepancy between the gross and pathological distal boundary of the tumour, which indicates how far the pathological tumour boundary extends beyond the gross boundary towards the distal resection stump. Details as to how ΔPM and ΔDM were calculated are shown in *Fig. 2*.

### Analysis

The last patient enrolled in this study underwent surgery 3 years before study completion. The primary endpoint of this study was defined as 3-year recurrence-free survival (RFS), which can serve as a surrogate marker for 5-year overall survival (OS)^19^.

First, optimal cut-off values for ΔPM and ΔDM were determined using time-dependent receiver operating characteristic (ROC) curve analysis of 3-year RFS. ROC curves were generated with ΔPM and ΔDM as continuous variables, and the cut-off values were defined as those with a maximum Youden index. Patients were divided into four groups: a short group, in which both ΔPM and ΔDM were shorter than the cut-off values; a long ΔPM group, in which ΔPM was longer and ΔDM was shorter than the cut-off values; a long ΔDM group, in which ΔPM was shorter and ΔDM was longer than the cut-off values; and a group in which both ΔPM and ΔDM were longer than the cut-off values (both-long group). Survival outcomes were compared between the four groups.

Second, all patients were divided into two groups: a short group, in which both ΔPM and ΔDM were shorter than the cut-off values; and a long group, in which ΔPM and/or ΔDM were longer than the cut-off values. Clinicopathological characteristics and survival outcomes were then compared between the two groups. Furthermore, to identify independent prognostic factors for RFS and OS, univariate and multivariate Cox regression analyses were performed. In the univariate analysis, ΔPM (> 8 *versus* ≤ 8 mm), ΔDM (> 3 *versus* ≤ 3 mm), ΔPM and ΔDM (long *versus* short group), age (≥ 75 *versus* < 75 years), sex (male *versus* female), clinical depth of tumour invasion (cT2–4 *versus* cT1), clinical lymph node metastasis (cN; positive *versus* negative), cOI (positive *versus* negative), growth type (infiltrative *versus* others), location (E, EG, or E = G *versus* GE, G), histological type (undifferentiated *versus* differentiated), pathological depth of tumour invasion (pT; T2–4 *versus* T1), and pathological lymph node metastasis (pN; positive *versus* negative) were input as variables. Multivariate Cox regression analysis included variables with *P* < 0.2 in the univariate analysis. Due to collinearity of cT and pT, cN and pN, as well as ΔPM, ΔDM, and the long group/short group, only pT, pN, and the long group/short group were included in multivariate analysis. pT and pN were selected over cT and cN because pathological staging is considered more accurate and reflective of actual tumour status than clinical staging. Similarly, long group/short group was selected over ΔPM and ΔDM because it integrates both margin parameters and better represents the prognostic impact.

Third, clinical prognostic factors influencing RFS in the long group were identified because it was assumed that ΔPM and ΔDM data could be obtained during surgery by analysis of intraoperative frozen sections (IFSs) and the information used. Which clinical factors were associated with poor survival outcomes was analysed in the long group using univariate and multivariate Cox regression analyses similar to the secondary analysis described above. For multivariate Cox regression analysis, pT and pN were not included, because information regarding pathological staging is not available during surgery.

### Statistical analysis

Data on the patients’ clinicopathological parameters, including tumour stage, were obtained by reviewing their medical charts. Statistical analyses, including Fisher’s exact test, time-dependent ROC analysis, log-rank test, and Cox regression analyses, along with data visualization, were performed using R version 4.2.3 (R Foundation for Statistical Computing, Vienna, Austria). Two-sided *P* < 0.050 was considered statistically significant.

## Results

### Patient characteristics, histograms, and cut-off levels for ΔPM and ΔDM

In all, 197 patients were finally enrolled in this study (*Fig. 1*). The background data of all patients are summarized in *Table 1*. A pathologically negative PM was observed in 191 patients in the initial transection of the oesophagus. A pathologically positive PM was observed in the remaining six patients, who underwent an additional transection to obtain a pathologically negative PM. No pathologically positive DM was observed in any patients during the initial transection of the stomach or duodenum. Histograms of ΔPM and ΔDM in all patients are shown in *Fig. S1*. Using time-dependent ROC analyses, the optimal cut-off values for ΔPM and ΔDM were determined to be 8 and 3 mm, respectively (*Fig. S2*).

**Table 1:** Comparison of clinicopathological features based on the extent of unexpected horizontal tumour spread

	Total (*n* = 197)	Short group (*n* = 125)	Long group (*n* = 72)	P*
**Clinical factors**				
Age (years)				
< 75	153 (77.7%)	97 (77.6%)	56 (78%)	1.000
≥ 75	44 (22.3%)	28 (22.4%)	16 (22%)	
Sex				
Male	156 (79.2%)	99 (79.2%)	57 (79%)	1.000
Female	41 (20.8%)	26 (20.8%)	15 (21%)	
cT status†				
T1	54 (27.4%)	38 (30.4%)	16 (22%)	0.248
T2–4	143 (72.6%)	87 (69.6%)	56 (78%)	
cN status†				
Negative	126 (64.0%)	79 (63.2%)	47 (65%)	0.878
Positive	71 (36.0%)	46 (36.8%)	25 (35%)	
Growth type†				
Superficial	70 (35.5%)	48 (38.4%)	22 (31%)	<0.001‡
Expansive	67 (34.0%)	51 (40.8%)	16 (22%)	
Infiltrative	60 (30.5%)	26 (20.8%)	34 (47%)	
Bormann classification†				
Type 4	1 (0.5%)	0	1 (1%)	0.365
Others	196 (99.5%)	125 (100.0%)	71 (99%)	
Location (Nishi’s classification)				
E, EG, E = G	40 (20.3%)	27 (21.6%)	13 (18%)	0.587
GE, G	157 (79.7%)	98 (78.4%)	59 (82%)	
cOI				
Negative	14 (7.1%)	12 (9.6%)	2 (3%)	0.088
Positive	183 (92.9%)	113 (90.4%)	70 (97%)	
**Surgical factors**				
Approach				
MIS	58 (29.4%)	43 (34.4%)	15 (21%)	0.052
Open	139 (70.6%)	82 (65.6%)	57 (79%)	
Procedure				
PG	68 (34.5%)	48 (38.4%)	20 (28%)	0.162
TG	129 (65.5%)	77 (61.6%)	52 (72%)	
**Pathological factors**				
Histopathological type				
Differentiated	127 (64.5%)	85 (68.0%)	42 (58%)	0.216
Undifferentiated	70 (35.5%)	40 (32.0%)	30 (42%)	
pT status†				
T1	57 (28.9%)	42 (33.6%)	15 (21%)	0.073
T2–4	140 (71.1%)	83 (66.4%)	57 (79%)	
pN status†				
Negative	96 (48.7%)	67 (53.6%)	29 (40%)	0.078
Positive	101 (51.3%)	58 (46.4%)	43 (60%)	
**Postoperative factors**				
Postoperative chemotherapy				
Without	122 (61.9%)	84 (67.2%)	38 (53%)	0.049
With	75 (38.1%)	41 (32.8%)	34 (47%)	
S-1 monotherapy	59 (30.0%)	33 (26.4%)	26 (36%)	0.196
Capecitabine/S-1 plus oxaliplatin	6 (3.1%)	2 (1.6%)	4 (6%)	0.194
S-1 plus cisplatin	4 (2.0%)	3 (2.4%)	1 (1%)	1.000
S-1 plus docetaxel	3 (1.5%)	1 (0.8%)	2 (3%)	0.555
UFT	2 (1.0%)	1 (0.8%)	1 (1%)	1.000
Unknown	1 (0.5%)	1 (0.8%)	0	1.000
**Long group types**				
ΔPM long			16 (22%)	
ΔDM long			42 (58%)	
Both long ΔPM and ΔDM			14 (20%)	

Values are expressed as *n* (%) unless otherwise stated. Patients were divided into two groups (short and long) according to the extent of unexpected horizontal tumour spread, measured as the discrepancy between gross and pathological margins proximally (ΔPM) and distally (ΔDM). The long group includes patients with long ΔPM, long ΔDM, or both long ΔPM and ΔDM. †According to the Japanese Classification of Gastric Carcinoma^4^. cT, clinical tumour depth; cN, clinical lymph node metastasis; E, tumours located in the proximal 2-cm segment of the oesophagogastric junction; G, tumours located in the distal 2-cm segment of the oesophagogastric junction; cOI, gross oesophageal invasion; MIS, minimum invasive surgery; PG, proximal gastrectomy; TG, total gastrectomy; pT, pathological tumour depth; pN, pathological lymph node metastasis; UFT, combination of tegafur and uracil. *Fisher’s exact test was used for statistical analyses, except ‡χ^2^ test.

### Survival outcomes of patients in the four groups based on ΔPM and ΔDM levels

The median follow-up period was 5.6 (range 0.04–14.7) years. Among the four groups (short, long ΔPM, long ΔDM, and both-long), the 3-year RFS rate was lowest (42.9%) in the both-long group (*Fig. S3a*). In contrast, the short group had the best survival outcome, with a 3-year RFS of 84.7%. The survival curve of the long ΔPM group was similar to that of the long ΔDM group, with intermediate survival outcomes relative to the short and both-long groups. Similar trends were observed for OS across the four groups (*Fig. S3b*).

### Clinicopathological features in two groups based on ΔPM and ΔDM levels

For subsequent analyses, patients were grouped into two categories: a short group (125) and a long group (72). The clinicopathological characteristics of these two groups are presented in *Table 1*. Tumours in the long group were more often infiltrative and associated with a higher rate of administration of adjuvant chemotherapy than in the short group.

### Survival outcomes in two groups based on ΔPM and ΔDM levels

**Fig. 3:**
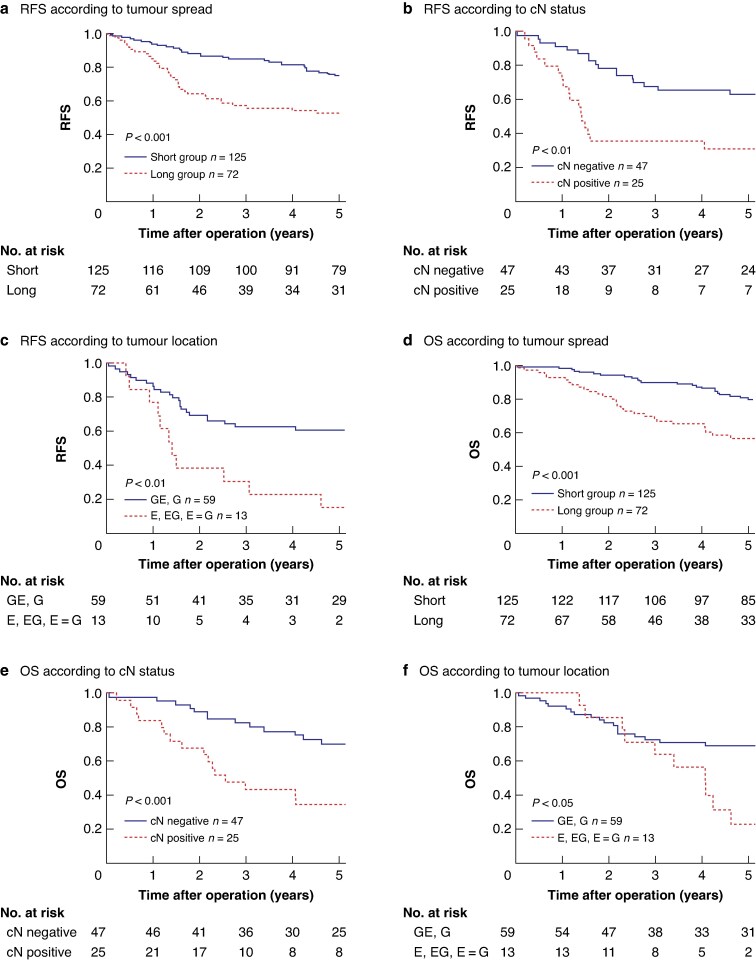
RFS and OS outcomes based on the extent of horizontal tumour spread in all patients (*n* = 197) and cN status in the long group (*n* = 72) **a** Kaplan–Meier curves of RFS in the long and short groups. Patients were divided into two groups (long and short) according to the optimal cut-off values for the extent of horizontal tumour spread, measured as the discrepancy between gross and pathological margins proximally (ΔPM) and distally (ΔDM). **b,c** Kaplan–Meier curves of RFS according to cN status (**b**) and tumour location (**c**) in the long group. **d** Kaplan–Meier curves of OS in the long and short groups. **e,f** Kaplan–Meier curves of OS according to cN status (**e**) and tumour location (**f**) in the long group. Survival was compared between groups using log-rank tests. RFS, recurrence-free survival; OS, overall survival; cN, clinical lymph node metastasis; E, tumours located in the proximal 2-cm segment of the oesophagogastric junction; G, tumours located in the distal 2-cm segment of the oesophagogastric junction.

Kaplan–Meier curves indicated that the long group had significantly worse RFS than the short group (*Fig. 3a*; 3-year RFS 56.9% *versus* 84.7%; *P* < 0.001). Similarly, OS was significantly worse in the long than short group (*Fig. 3c*; 5-year OS 57.7% *versus* 80.3%; *P* < 0.001). In the long group, 25 patients (34.7%) experienced recurrence, compared with 21 patients (16.8%) in the short group (*Table S1*). Lung metastases were more frequently identified in the long group than in the short group.

### Univariate and multivariate analyses of risk factors for RFS and OS

Univariate analysis consistently revealed that ΔPM, ΔDM, and the long group were predictors of both RFS and OS. After excluding factors with collinearity, multivariate analyses identified the long group, pT status, and pN status as independent risk factors for RFS (*Table 2*), and the long group as an independent risk factor for OS (*Table S2*).

**Table 2:** Univariate and multivariate Cox regression analyses of factors related to recurrence-free survival (*n* = 197)

	Univariate analysis		Multivariate analysis	
	HR	*P*	HR	*P*
**ΔPM, mm**				
≤8	1.00 (Reference)			
>8	2.24 (1.27–3.95)	0.005		
**ΔDM, mm**				
≤3	1.00 (Reference)			
>3	2.26 (1.37–3.71)	0.001		
Unexpected horizontal tumour spread*				
Short group	1.00 (Reference)		1.00 (Reference)	
Long group	2.45 (1.50–4.01)	<0.001	2.05 (1.23–3.42)	0.006
**Age, years**				
<75	1.00 (Reference)			
≥75	0.94 (0.52–1.70)	0.835		
**Sex**				
Female	1.00 (Reference)			
Male	1.48 (0.76–2.91)	0.252		
**cT status†**				
T1	1.00 (Reference)			
T2–T4	3.70 (1.69–8.11)	0.001		
**cN status†**				
Negative	1.00 (Reference)			
Positive	2.94 (1.79–4.82)	<0.001		
**cOI**				
Negative	1.00 (Reference)		1.00 (Reference)	
Positive	2.65 (0.65–10.84)	0.175	1.05 (0.25–4.43)	0.952
**Growth type†**				
Superficial or expansive	1.00 (Reference)		1.00 (Reference)	
Infiltrative	2.99 (1.83–4.89)	<0.001	1.35 (0.78–2.33)	0.279
**Location‡**				
GE, G	1.00 (Reference)			
E, EG, or E = G	1.36 (0.77–2.40)	0.287		
**Histological type**				
Differentiated	1.00 (Reference)			
Undifferentiated	0.89 (0.53–1.50)	0.666		
**pT status†**				
T1	1.00 (Reference)		1.00 (Reference)	
T2–T4	4.87 (2.10–11.28)	<0.001	2.76 (1.12–6.77)	0.027
**pN status†**				
Negative	1.00 (Reference)		1.00 (Reference)	
Positive	3.68 (2.09–6.48)	<0.001	2.52 (1.38–4.62)	0.003

Values in parentheses are 95% confidence intervals. *Patients were divided into two groups (short and long) according to the extent of unexpected horizontal tumour spread, measured as the discrepancy between gross and pathological margins proximally (ΔPM) and distally (ΔDM). The long group includes patients with long ΔPM, long ΔDM, or both long ΔPM and ΔDM. †According to the Japanese Classification of Gastric Carcinoma^4^. ‡According to Nishi’s classification^4^. HR, hazard ratio; cT, clinical tumour depth; cN, clinical lymph node metastasis; cOI, clinical oesophageal invasion; E, tumours located in the proximal 2-cm segment of the oesophagogastric junction; G, tumours located in the distal 2-cm segment of the oesophagogastric junction; pT, pathological tumour depth; pN, pathological lymph node metastasis.

### Subgroup analyses of survival outcomes

Forest plots with hazard ratios for the long and short groups for RFS and OS according to different clinicopathological factors are shown in *Fig. S4*. Both RFS and OS of the long group were significantly worse in most subgroups.

### Clinical factors associated with poor survival in the long group

Univariate and multivariate analyses of predictors in the long group revealed that cN positivity and location (E, EG, E = G) were independent risk factors for poor RFS (*Table 3*) and OS (*Table S3*). Kaplan–Meier curves based on cN status and tumour location in the long group were also analysed. The 3-year RFS and 5-year OS rates were 36 and 35%, respectively, in patients with cN positivity (*Fig. 3b*,*e*). Kaplan–Meier curves stratified by cN negative, cN number < 3, and cN number ≥ 3 showed that prognosis worsened as the number of clinically suspected lymph node metastases increased (*Fig. S5*). The 3-year RFS and 5-year OS rates were 31 and 18%, respectively, in patients with tumours locations of E, EG, or E = G (*Fig. 3c*,*f*).

**Table 3:** Analysis of factors related to recurrence-free survival in patients with long unexpected horizontal tumour spread (*n* = 72)

	Univariate analysis			Multivariate analysis		
		HR	*P*		HR	*P*
**Age (years)**						
<75	1.00	(Reference)				
≥75	0.78	(0.34–1.79)	0.557			
**Sex**						
Female	1.00	(Reference)				
Male	1.36	(0.56–3.28)	0.497			
cT status*						
T1	1.00	(Reference)		1.00	(Reference)	
T2–T4	2.15	(0.83–5.56)	0.115	1.16	(0.37–3.67)	0.800
cN status*						
Negative	1.00	(Reference)		1.00	(Reference)	
Positive	2.90	(1.47–5.72)	0.002	2.71	(1.26–5.82)	0.010
**cOI**						
Negative	1.00	(Reference)				
Positive	1.21	(0.17–8.84)	0.852			
Growth type*						
Superficial or expansive	1.00	(Reference)		1.00	(Reference)	
Infiltrative	1.90	(0.96–3.77)	0.066	1.43	(0.64–3.20)	0.378
**Location†**						
GE, G	1.00	(Reference)		1.00	(Reference)	
E, EG, or E = G	3.12	(1.51–6.45)	<0.001	3.49	(1.65–7.35)	0.001
**Histological type**						
Differentiated	1.00	(Reference)				
Undifferentiated	0.84	(0.42–1.69)	0.635			
pT status*						
T1	1.00	(Reference)				
T2–T4	2.58	(0.91–7.34)	0.075			
pN status*						
Negative	1.00	(Reference)				
Positive	3.88	(1.68–8.94)	0.001			

Values in parentheses are 95% confidence intervals. *According to the Japanese Classification of Gastric Carcinoma^4^. †According to Nishi’s classification^4^. HR, hazard ratio; cT, clinical tumour depth; cN, clinical lymph node metastasis; cOI clinical oesophageal invasion; E, tumours located in the proximal 2-cm segment of the oesophagogastric junction; G, tumours located in the distal 2-cm segment of the oesophagogastric junction; pT, pathological tumour depth; pN, pathological lymph node metastasis.

## Discussion

In this retrospective study, the oncological significance of unexpected horizontal tumour spread, measured as ΔPM and ΔDM, in OGJ cancer was investigated. The key results demonstrated the prognostic impact of these parameters, with optimal cut-off values of 8 and 3 mm for ΔPM and ΔDM, respectively. The long group was significantly associated with both poor survival outcomes and postoperative recurrence. Furthermore, patients in the both-long group and those with cN positivity or an oesophageal lesion (E, EG, E = G) in the long group had markedly poorer survival outcomes. These findings provide new insights into optimizing surgical strategies and refining prognostic assessments for OGJ cancer.

Unexpected horizontal tumour spread can extend towards both the proximal and distal margins. In this study, the histograms for both ΔPM and ΔDM almost show a normal distribution, consistent with previous reports^8–11,18^, suggesting that these phenomena may reflect a natural biological process. Moreover, consistent with the cut-off value for ΔPM previously reported in gastric cancer or OGJ cancer^12^, the optimal cut-off value for ΔPM in OGJ cancer in the present study was identified as 8 mm by time-dependent ROC analysis. These results support that ΔPM and ΔDM are reliable indicators of unexpected horizontal tumour spread, providing valuable prognostic information.

In addition, in analyses of the four groups, survival outcomes were the best for the group with both short margins, whereas they were worst for the both-long group. The long ΔPM and long ΔDM groups had intermediate and comparable survival outcomes. These findings indicate a quantitative association between unexpected horizontal tumour spread and survival outcomes. Notably, both proximal and distal spread have similar oncological impacts. Therefore, categorizing patients into two groups based on the length of the unexpected horizontal tumour spread is a logical approach.

Comparative analysis between the short and long groups revealed marked differences. The long group had more aggressive tumour characteristics, such as infiltrative growth types and deeper tumour invasion, which were associated with higher recurrence rates and poorer outcomes. Multivariate analyses confirmed the long group as an independent risk factor for both RFS and OS. These findings indicate that ΔPM and ΔDM may serve as surrogate markers for tumour aggressiveness, enabling the identification of high-risk patients who may benefit from more intensive adjuvant treatments or vigilant postoperative surveillance.

Importantly, information regarding ΔPM and ΔDM may be used during surgery on a limited basis when the initial transected margin is pathologically positive by IFS analysis despite a gross PM length of > 8 mm and/or a gross DM length of > 3 mm in PG, which indicates a ΔPM > 8 mm and/or ΔDM > 3 mm, belonging to the long group. In the present study, patients in the long group, especially in the both-long group, or those with cN positivity in the long group were found to have a poor survival outcome, with 3-year RFS rates of 42.9 and 36%, respectively. Furthermore, patients in the long group with tumours locations of E, EG, or E = G also had notably poor survival outcomes. Most of these patients experienced recurrence within 2 years, suggesting rapid tumour progression with poor outcomes even when complete local resection was achieved. This information could serve as a useful reference when considering additional resections during IFS analysis if ΔPM > 8 mm and/or ΔDM > 3 mm is identified. Assessing ΔPM and ΔDM in combination with clinical factors could enhance intraoperative decision-making in clinical practice.

Interestingly, this study found a strong association between the long group and distant metastases, particularly lung metastases, rather than local recurrence. This suggests that ΔPM and ΔDM reflect not only local tumour extension but also systemic tumour biology, including metastatic potential. These findings align with the concept of minimal residual disease, often detected through liquid biopsy techniques like circulating tumour DNA analyses^20,21^. Although circulating tumour DNA analysis is a powerful method for the detection of minimal residual disease, its high cost and technical demands limit its accessibility. However, ΔPM and ΔDM are practical and cost-effective parameters that can be readily evaluated during routine surgical procedures. The association between unexpected horizontal tumour spread and minimal residual disease requires further validation, and future research is needed to clarify the underlying mechanisms.

Despite its strengths, this study has limitations. First, as a single-centre retrospective study, the generalizability of the findings may be limited. However, it would be difficult to conduct a prospective study, and the disadvantages of the retrospective nature of the present study may be minimal. Second, in the pathological examination, whole tumour sections were not always obtained as part of routine practice. Thus, the longest pathological extension could exist at a site where a section was not taken for pathological evaluation, potentially resulting in overestimation of the pathological PM length. Third, upfront surgery remains the main treatment for OGJ cancer in East Asia, including Japan. This study excluded patients who underwent preoperative chemotherapy, a standard treatment in many Western countries, to avoid confounding effects on tumour margins. Thus, the information from this study is not directly applicable to Western daily practice, especially because tumours of the OGJ are more often operated on via a transthoracic approach (for example Ivor Lewis oesophagectomy). Investigating the prognostic impact of unexpected horizontal tumour spread after preoperative chemotherapy will be an important subject for future research.

In conclusion, this study provides robust evidence that unexpected horizontal tumour spread in OGJ cancer significantly impacts recurrence and survival outcomes. Assessing ΔPM and ΔDM is not only valuable for identifying high-risk patients and predicting prognosis but can also serve as a reference for guiding additional resections during IFS analysis when combined with other predictive factors.

## Supplementary Material

zraf119_Supplementary_Data

## Data Availability

The data sets used and/or analysed during this study are available from the corresponding author upon reasonable request.
