# Supplementary material for: Oncological impact of unexpected horizontal tumour spread in oesophagogastric junction cancer
Source: BJS Open. 2025 Oct 7;9(5):zraf119. doi: 10.1093/bjsopen/zraf119 (PMC12502906; doi:10.1093/bjsopen/zraf119)
Supplement: zraf119_Supplementary_Data [file zraf119_supplementary_data.docx]

**Oncological impact of unexpected horizontal tumor spread in esophagogastric junction cancer**

Qingjiang Hu, Manabu Ohashi, Motonari Ri, Rie Makuuchi, Tomoyuki Irino, Masaru Hayami, Takeshi Sano, Souya Nunobe

**Affiliations:**

Department of Gastroenterological Surgery, Cancer Institute Hospital, Japanese Foundation for Cancer Research, Tokyo, Japan

**Corresponding Author:**

Manabu Ohashi, Department of Gastroenterological Surgery, Cancer Institute Hospital, Japanese Foundation for Cancer Research, 3-8-31 Ariake, Koto-ku, Tokyo, 135-8550, Japan

Email: manabu.ohashi@jfcr.or.jp

Tel: +81-03-3520-0111, Fax: +81-03-3520-0141

**Supplementary Materials - Index**

| **Supplementary Figures and Tables** | Page |
| --- | --- |
| Figure S1. | 2 |
| Figure S2. | 3 |
| Figure S3. | 4 |
| Figure S4. | 5 |
| Figure S5. | 6 |
| Table S1. | 7 |
| Table S2. | 8, 9 |
| Table S3. | 10 |

**Figure S1. Distribution of ΔPM and ΔDM (n = 197).** The horizontal axes of the histograms represent the range of ΔPM and ΔDM lengths, and the vertical axes represent the number of patients. Each range does not include the upper boundary value. a. Histogram of ΔPM. b. Histogram of ΔDM.


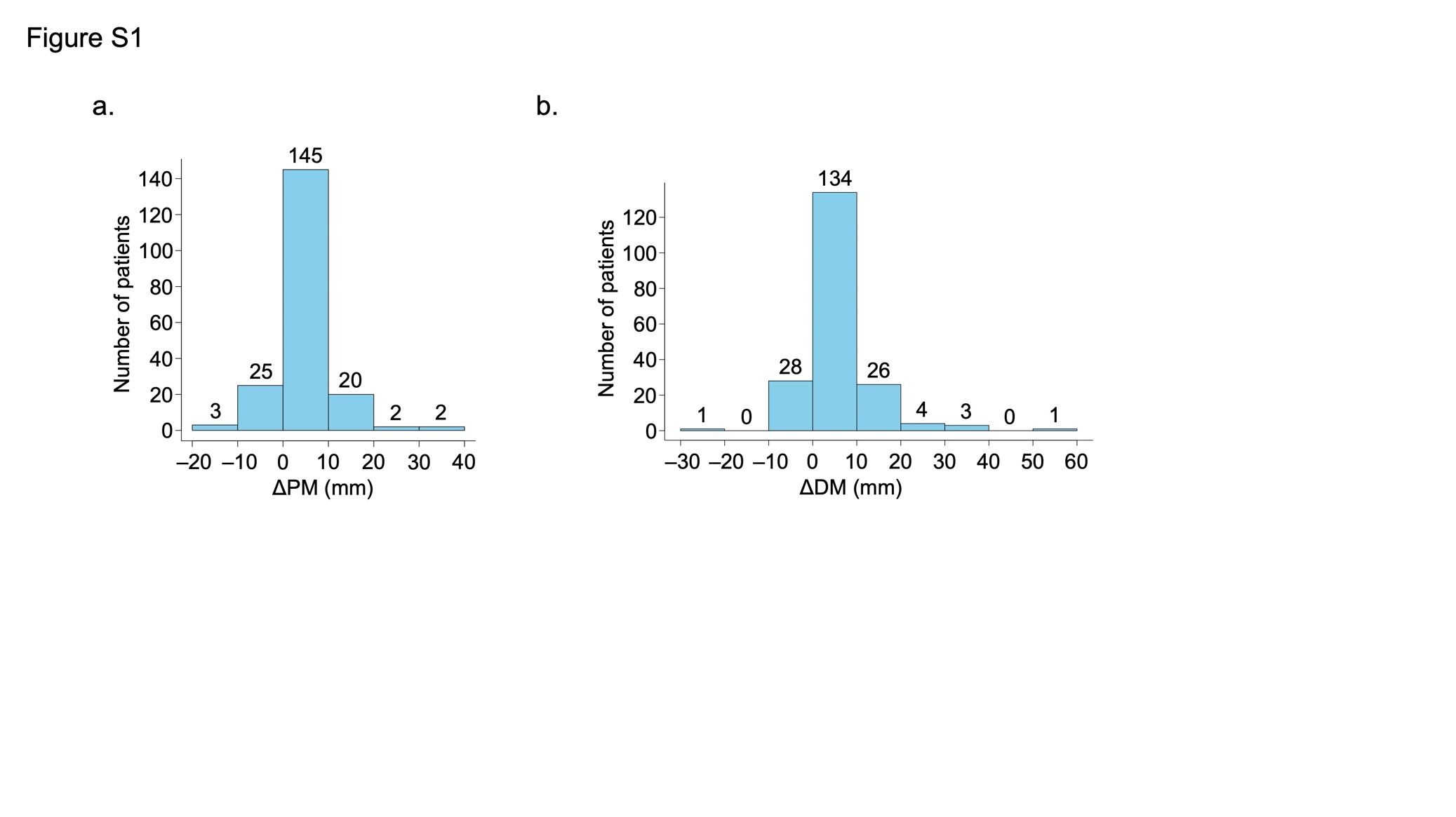


b

a

**Figure S2.** **Time-dependent ROC analyses curve used to determine the optimal cutoff values for ΔPM and ΔDM in predicting 3-year RFS in all patients (n = 197).** a. The optimal cutoff of ΔPM was 8 mm with an AUC of 0.58. b. The optimal cutoff of ΔDM was 3 mm with an AUC of 0.63. ROC, receiver operating characteristic; RFS, recurrence-free survival; AUC, area under the curve.

**
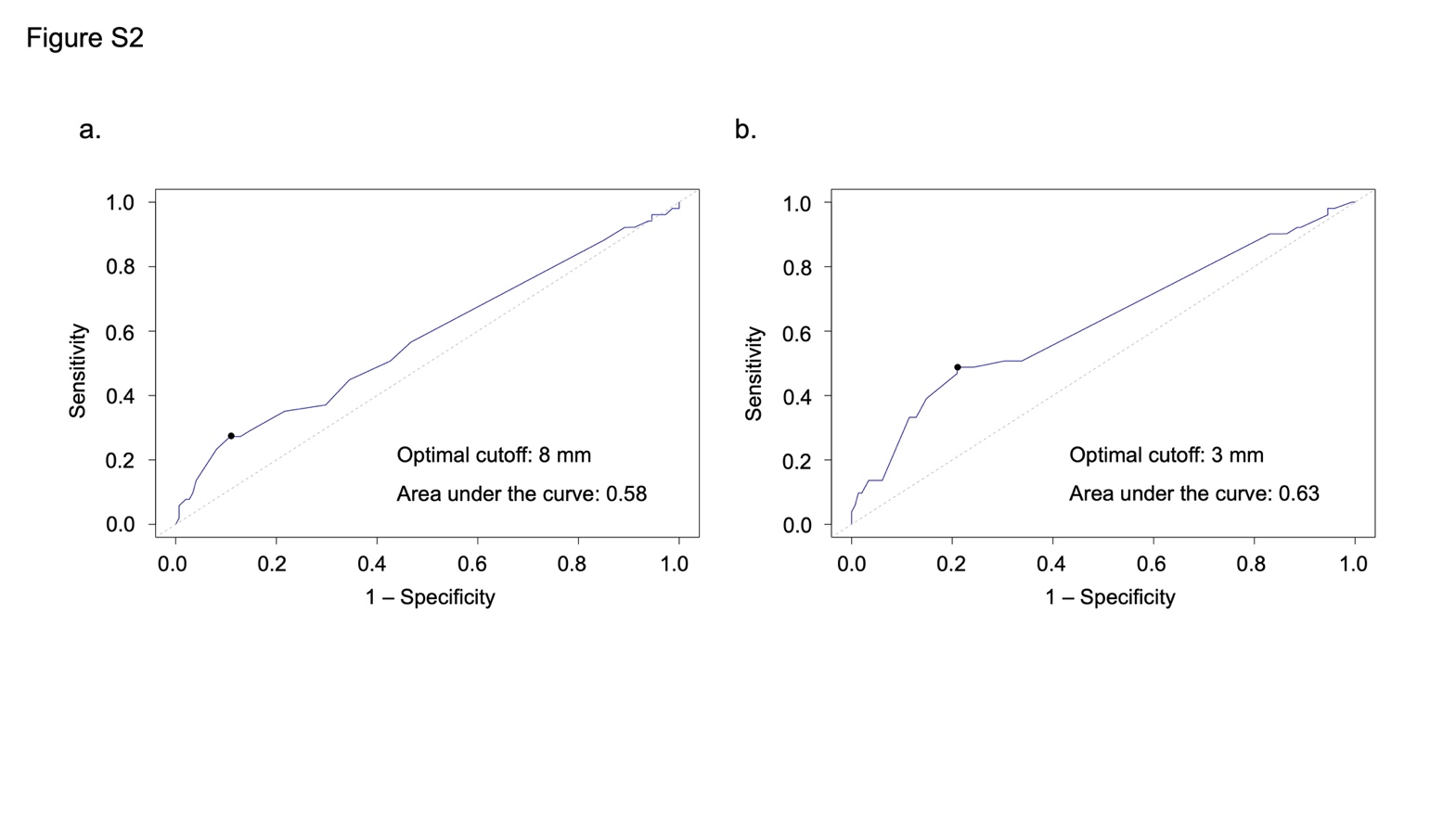
**

a

b

**Figure S3. Survival outcomes based on ΔPM and ΔDM levels in all patients (n = 197).** Patients were divided into four groups according to the optimal cutoff values for ΔPM and ΔDM. Kaplan-Meier curves were used to compare survival outcomes between these groups. a. Kaplan-Meier curves for RFS across the four groups. b. Kaplan-Meier curves for OS across the four groups. RFS, recurrence-free survival; OS, overall survival.

**
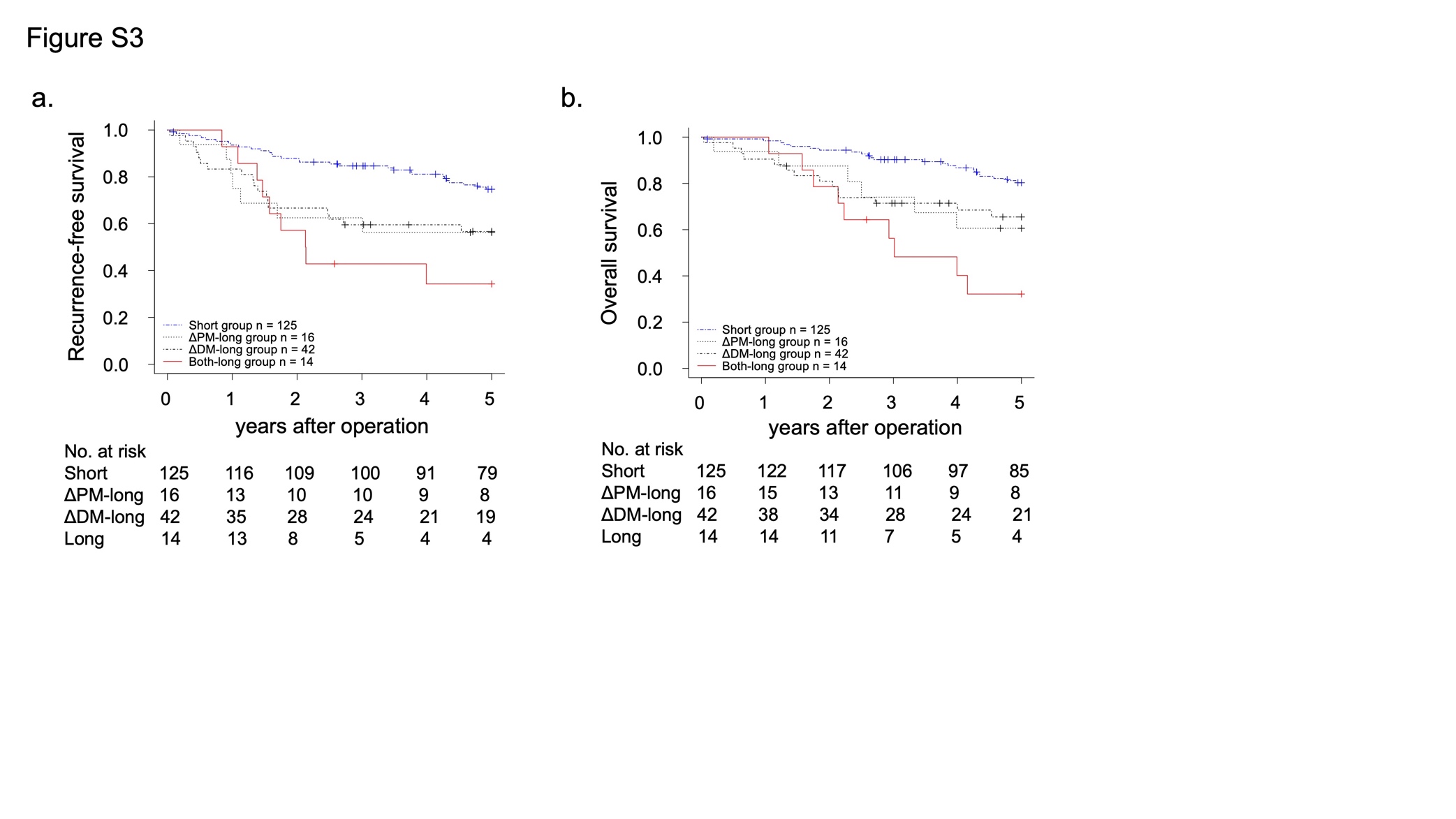
**

a

b

**Figure S4. Subgroup analyses of survival outcomes based on clinicopathological factors in all patients (n = 197).** Forest plots showing the HRs of long groups with 95% CI, derived from univariate Cox regression analysis within each subgroup. a. Forest plot displaying HRs for RFS. b. Forest plot displaying HRs for OS. HR, hazard ratio; CI, confidence interval; RFS, recurrence-free survival; OS, overall survival.

**
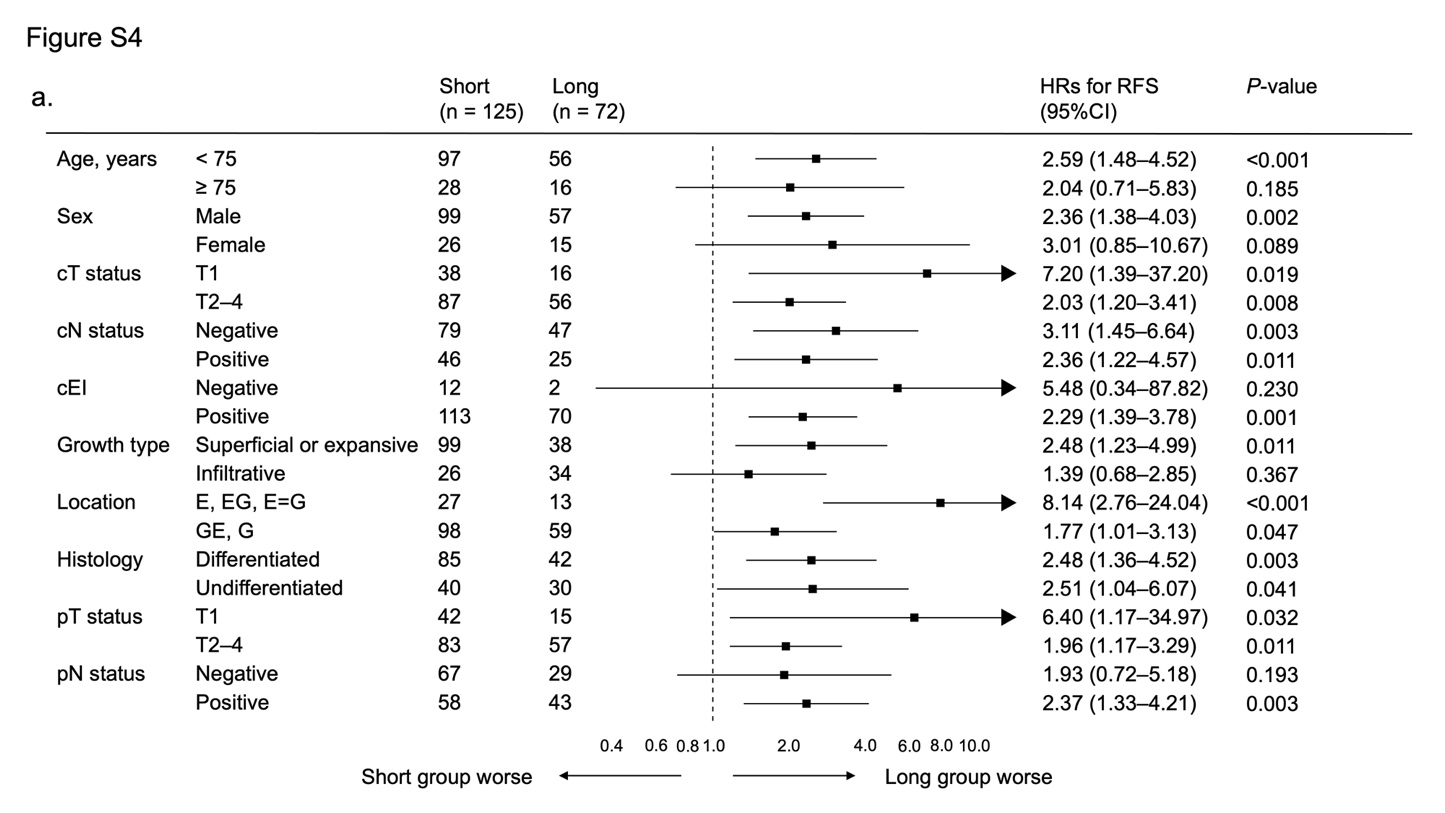
**

a

**
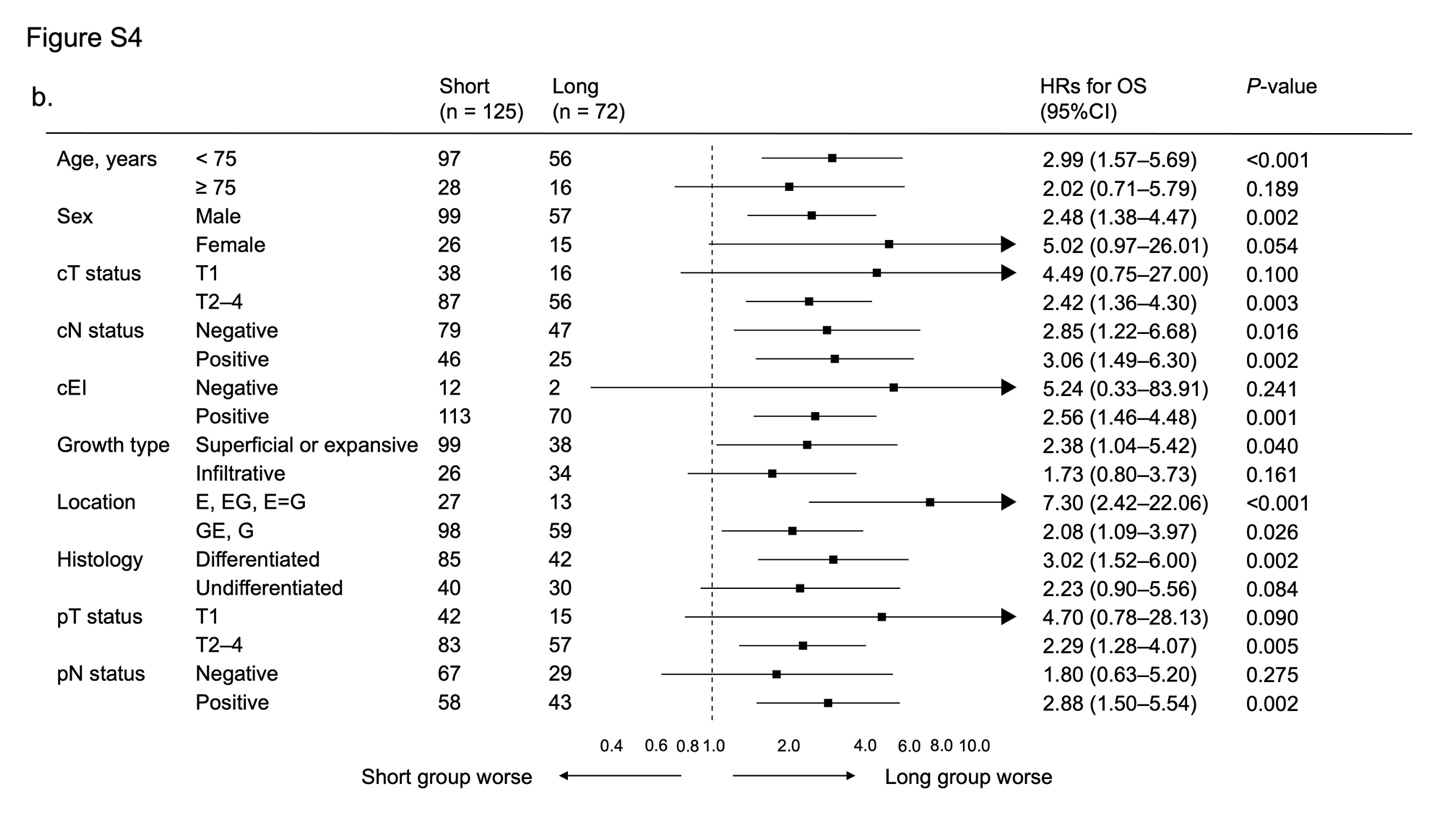
**

b

**Figure S5. Survival outcomes based on cN status in the long group (n = 72).** Patients were divided into three groups (cN negative, cN number < 3, and cN number ≥ 3) based on the number of clinically suspected lymph node metastases. Kaplan-Meier curves were used to compare survival outcomes between these groups. a. Kaplan-Meier curves for RFS based on cN status. b. Kaplan-Meier curves for OS based on cN status. RFS, recurrence -free survival; OS, overall survival.

**
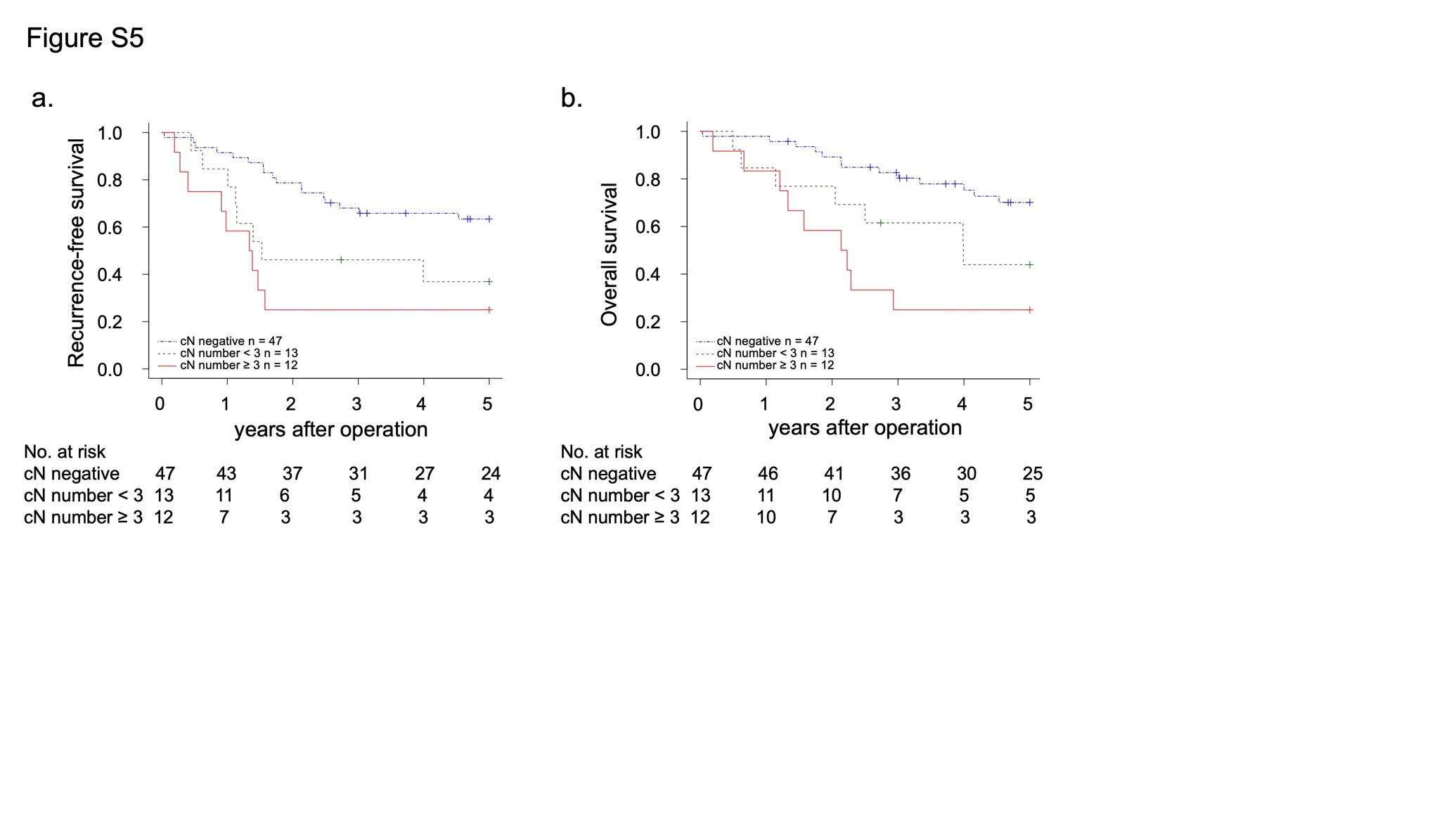
**

a

b

**Table S1.** Comparison of recurrent types based on ΔPM and ΔDM levels

| Factors | Short group | Long group | *P*-value |
| --- | --- | --- | --- |
|  | (n = 125) | (n = 72) |  |
| Recurrence  With  Without | 21 (16.8)  104 (83.2) | 25 (35)  47 (65) | 0.005 |
| Metastatic site | | | |
| Peritoneum | 5 (4.0) | 4 (6) | 0.727 |
| Lung | 4 (3.2) | 8 (11) | 0.033 |
| Lymph node | 11 (8.8) | 8 (11) | 0.622 |
| Bone | 2 (1.6) | 2 (3) | 0.624 |
| Liver | 10 (8.0) | 5 (7) | 1.000 |
| Local | 1 (0.8) | 1 (1) | 1.000 |

Values are expressed as n (%).

Fisher’s exact test was used for statistical analyses.

Short group, both ΔPM and ΔDM were shorter than the cutoff values.

Long group, patients with ΔPM-long, ΔDM-long or both-long.

**Table S2.** Univariate and multivariate Cox regression analyses for OS (n = 197)

| Factors | Univariate analysis | | | Multivariate analysis | | |
| --- | --- | --- | --- | --- | --- | --- |
|  | HR | 95%CI | *P*-value | HR | 95%CI | *P*-value |
| ΔPM, mm (> 8/≤ 8) | 2.78 | 1.52–5.07 | <0.001 |  |  |  |
| ΔDM, mm (> 3/≤ 3) | 2.46 | 1.42–4.26 | 0.001 |  |  |  |
| Long group/short group | 2.72 | 1.57–4.71 | <0.001 | 2.23 | 1.26–3.93 | 0.006 |
| Age, years (≥ 75/< 75) | 1.33 | 0.72–2.45 | 0.363 |  |  |  |
| Sex (male/female) | 1.74 | 0.79–3.86 | 0.172 | 1.65 | 0.74–3.68 | 0.222 |
| cT status (cT2–4/T1) ^a^ | 3.98 | 1.58–10.00 | 0.003 |  |  |  |
| cN status (positive/negative) ^a^ | 2.87 | 1.65–4.98 | <0.001 |  |  |  |
| cEI (positive/negative) | 2.03 | 0.49–8.35 | 0.325 |  |  |  |
| Growth type (infiltrative/  superficial or expansive) ^a^ | 3.56 | 2.06–6.16 | <0.001 | 1.78 | 0.96–3.32 | 0.068 |
| Location (E, EG, E=G/GE, G) ^b^ | 1.54 | 0.85–2.81 | 0.158 | 1.60 | 0.86–2.95 | 0.135 |
| Histological type (undifferentiated /differentiated) | 1.12 | 0.64–1.96 | 0.701 |  |  |  |
| pT status (pT2–4/T1) ^a^ | 4.45 | 1.77–11.20 | 0.001 | 2.34 | 0.86–6.42 | 0.098 |
| pN status (Positive/Negative) ^a^ | 3.13 | 1.69–5.77 | <0.001 | 1.92 | 0.98–3.76 | 0.056 |

OS, overall survival; HR, hazard ratio; CI, confidence interval; cT, clinical tumor depth; cN, clinical lymph node metastasis; cEI clinical esophageal invasion; pT, pathological tumor depth; pN, pathological lymph node metastasis; Und, undifferentiated type; Dif, differentiated type.

Data were analyzed using Cox regression analysis.

Short group, both ΔPM and ΔDM were shorter than the cutoff values.

Long group, patients with ΔPM-long, ΔDM-long or both-long.

^a^ According to the Japanese Classification of Gastric Carcinoma.

^b^ According to Nishi’s classification.

**Table S3.** Univariate and multivariate Cox regression analyses for OS in patients of the long group (n = 72)

| Factors | Univariate analysis | | | Multivariate analysis | | |
| --- | --- | --- | --- | --- | --- | --- |
|  | HR | 95%CI | *P*-value | HR | 95%CI | *P*-value |
| Age, years (≥ 75/< 75) | 1.11 | 0.47–2.60 | 0.812 |  |  |  |
| Sex (male/female) | 1.35 | 0.51–3.53 | 0.546 |  |  |  |
| cT status (cT2–4/T1) ^a^ | 2.93 | 0.89–9.67 | 0.078 | 1.38 | 0.33–5.69 | 0.659 |
| cN status (positive/negative) ^a^ | 3.24 | 1.55–6.76 | 0.002 | 2.52 | 1.13–5.65 | 0.024 |
| cEI (positive/negative) | 0.89 | 0.12–6.52 | 0.906 |  |  |  |
| Growth type (infiltrative/  Superficial or expansive) ^a^ | 2.45 | 1.14–5.27 | 0.022 | 1.65 | 0.68–4.01 | 0.265 |
| Location (E, EG, E=G/GE, G) ^b^ | 2.46 | 1.14–5.31 | 0.022 | 2.33 | 1.06–5.12 | 0.034 |
| Histological type (undifferentiated /differentiated) | 0.95 | 0.45–2.02 | 0.901 |  |  |  |
| pT status (pT2–4/T1) ^a^ | 2.84 | 0.86–9.40 | 0.087 |  |  |  |
| pN status (Positive/Negative) ^a^ | 3.61 | 1.46–8.91 | 0.005 |  |  |  |

Data were analyzed using Cox regression analysis.

OS, overall survival; HR, hazard ratio; CI, confidence interval; cT, clinical tumor depth; cN, clinical lymph node metastasis; cEI clinical esophageal invasion.
^a^ According to the Japanese Classification of Gastric Carcinoma.

^b^ According to Nishi’s classification.
